# Long-term survival of hepatocellular carcinoma after percutaneous radiofrequency ablation guided by ultrasound

**DOI:** 10.1186/s12957-017-1189-1

**Published:** 2017-07-05

**Authors:** Weimin Zhang, Erping Luo, Jianhe Gan, Xiaomin Song, Zuowei Bao, Huiping Zhang, Minhua Chen

**Affiliations:** 1 Department of Infectious, The First Affiliated Hospital of Soochow University, No. 188 Shizi Street, Suzhou, 215006 Jiangsu Province China; 2 Department of Ultrasound, Xuhui Centre Hospital, Fudan University, 200031 Shanghai, China; 3 Department of Ultrasound, The Third People’s Hospital of Changzhou, Changzhou, 213001 Jiangsu Province China; 4 Department of Ultrasound, The People’s Hospital of Maanshan, Maanshan, 243000 Anhui Province China; 5 Cancer Hospital of Beijing, 100142 Beijing, China

**Keywords:** Hepatocellular carcinoma, Radiofrequency ablation, Survival, Recurrence, Risk factors

## Abstract

**Background:**

The risk factors for recurrence and death after radiofrequency ablation (RFA) for hepatocellular carcinoma (HCC) remain poorly known. This study was aimed to study the 10-year overall survival (OS) of HCC treated by ultrasound (US)-guided RFA and the risk factors for recurrence and death.

**Methods:**

Between June 2005 and June 2016, 1000 patients with HCC treated by US-guided RFA at 4 hospitals in China; among them, 525 patients met the criteria for radical ablation and 410 had high AFP levels before RFA treatment. Clinical and biochemical factors were tested for association with recurrence and survival. Patients were divided into the recurrence (*n* = 348) and no recurrence groups (*n* = 62).

**Results:**

The 5- and 10-year survival rates were 66 and 35%, respectively. Tumor size (HR = 1.36, 95% CI 1.12–1.65), albumin levels (HR = 0.76, 95% CI 0.65–0.91), prothrombin time (HR = 2.18, 95% CI 1.54–3.10), and α-fetoprotein levels (HR = 1.13, 95% CI 1.00–1.26) were independently associated with mortality after RFA for HCC. Tumor size (HR = 1.27, 95% CI: 1.15–1.40), HBV-DNA (HR = 7.70, 95% CI 3.57–16.63), AFP levels before treatment (HR = 2.172, 95% CI 1.256–3.756, *P* = 0.006), and AFP response (HR = 4.722, 95% CI 1.053–21.184, *P* = 0.0427) were independently associated with the risk of recurrence of HCC after RFA. The median survival of the patients with and without recurrence after RFA was 54 (95% CI 45–58) and 62 (95% CI 48–80) months, respectively (log-rank, *P* = 0.04).

**Conclusions:**

Tumor size, albumin, prothrombin time, and α-fetoprotein levels were independently associated with mortality after US-guided RFA for HCC, while tumor size and HBV-DNA were independently associated with recurrence. Patients with recurrence had a poorer survival compared with those without.

## Background

Hepatocellular carcinoma (HCC) is the fifth most common malignant tumor in the world [1], and the third leading cause of cancer death worldwide [2]. Because of long-term virus infection, impaired liver function, fibrosis, tumor location, and tumor multifocality, the overall resectability rate for such lesions is <20% [3]. Similarly, surgical resection carries a significant risk of morbidity and mortality, with a risk of complications as high as 50–81% [4–6]. According to the EASL Guidelines on HCC [6], surgical resection, transplantation, and local standardized thermal ablative therapy have been considered the gold standard for treatment of HCC.

For selected patients, radiofrequency ablation (RFA) is an alternative approach that achieves survival rates similar to those obtained with surgery, but with fewer risks [7–11]. Indeed, RFA is minimally invasive, can be performed with local anesthesia, and have a low complication rate. Therefore, RFA has a good cost-effectiveness compared with other approaches and maximizes the preservation of the surrounding liver parenchyma while minimizing hospitalization [12]. In general, RFA is performed on single lesions <5 cm in diameter or ≤3 lesions <3 cm in largest diameter measured by contrast-enhanced ultrasound (CEUS), Child-Pugh class A or B, and ECOG 0, based on current guidelines from the AASLD [12].

However, according to the literature, the recurrence rate could be higher with RFA than with surgery [8]. Shiina et al. [5] reported a 10-year survival rate of 27.3% after RFA. They also showed that anti-HCV, Child-Pugh class, platelet count, tumor size, tumor number, serum α-fetoprotein (AFP) levels, and serum des-gamma-carboxy-prothrombin (DCP) levels were significantly related to distant recurrence [5]. Nevertheless, the risk factors for recurrence and death after RFA for HCC remain poorly known. In addition, data specific to the Chinese population are lacking and, compared with western countries and Japan, the epidemiology of HCC in China is characterized by a high rate of chronic hepatitis B virus (HBV) infection [13].

Therefore, the present study summarizes 1000 Chinese patients with HCC treated by ultrasound-guided RFA over 11 years. Among them, 525 patients were considered as having received curative RFA.

## Methods

### Study design

This was a retrospective study of 1000 consecutive patients with HCC treated at the First Affiliated Hospital of Soochow University (Jiangsu, China), the Changzhou Third People’s Hospital (Jiangsu, China), the Xuhui District Center Hospital (Shanghai, China), and the People’s Hospital of Maanshan (Anhui, China) between June 2005 and June 2016. This study was approved by the Ethics Committee of the People’s Hospital of Maanshan.

### Patients

Inclusion criteria were (1) histological diagnosis of HCC; (2) focal lesion with arterial enhancement and portal venous washout on CEUS, computed tomography (CT), or magnetic resonance imaging (MRI); and (3) complete ablation of the target lesions. Exclusion criteria were (1) portal vein tumor thrombus; (2) tumor margin close to portal vein branch by <1 mm; or (3) distant metastases. Finally, 525 patients met the criteria.

### Pretreatment evaluation

The pretreatment assessment of each patient included renal and liver function, complete blood count (CBC), prothrombin time, ascites, AFP, HBV DNA, and electrocardiogram. Pretreatment image examination included ultrasound, CEUS, CT, and/or MRI of the abdomen.

This study was in the field of interventional ultrasound. Therefore, according to the BCLC diagnostic criteria of HCC, patients with 2 typical vascular imaging presentations, or with 1 dynamic typical imaging presentation and AFP >400 ng/ml or confirmed by histopathological examinations were diagnosed with HCC. As the RFA was performed under ultrasound guidance, ultrasound was chosen as the main method to determine the maximum diameter of the tumor, locations, sizes, shapes, and extensions.

### RFA

The Esaote Mylab 90 (ESAOTE S.p.A., Milan, Italy) and ACUSON S2000 (Siemens, Erlangen, Germany) were used for guidance, using the Sonovue ultrasound contrast agent (Bracco S.p.A., Milan, Italy). Intramuscular injection of 50 ml of dolantin and 1 ampoule of diazepam was performed before the operation. Fentanyl was used for sedation and analgesia during the operation under monitoring. Lidocaine was used for subcutaneous injection and liver capsule injection. The patients were placed in the supine position and were asked to cooperate with the clinicians during the puncture by breathing appropriately.

Preoperative planning including evaluation of all comparative imaging studies and careful ultrasound scan was performed to identify the tumor size and target access. A Valleylab Cooltip (Valleylab, boulder, Co., USA) 17-gauge single electrode with a 3-cm effective tip was used for lesions <2 cm in diameter. For tumors >2 cm, a 15.5-gauge Olympus Celon bipolar electrode T30 + T30/T40 + T40 (Olympus, Teltow, Germany) was used, while being sure that the electrode tips distance inside the tumor was <25 mm [14–17]. Attention was paid to the heat-sink effect [18] during perivascular lesion ablation. The Valleylab Cooltip device was used to ablate 1 lesion for 12 min. For the Olympus Celon bipolar electrode the ablation time was estimated according to the impedance, total Joule of ablation, and intraoperative image changes. All the procedures were performed according to the manufacturer’s protocols.

### Follow-up

Patients underwent ultrasound or CEUS of the liver 1 month after RFA, as well as AFP and liver biochemistry assessment. Thereafter, surveillance of the ablated zone and residual liver was assessed by ultrasound and CEUS every 3 months.

If AFP continued to increase during follow-up, MRI or CT examination was performed. Positron emission tomography (PET) was used for some patients. Patients showing changed in the RFA area, unclear boundaries, nodular enhancement in the arterial phase, and new nodules were considered with intrahepatic recurrence of HCC. The follow-up for the evaluation of local treatment effectiveness of RFA was performed according to the revised response evaluation criteria in solid tumors (mRECIST). Of the 525 cases of HCC patients with RFA treatment, 1 month after the procedure and immediate postprocedure, radiographic modified RECIST Criterion for evaluation of short-term efficacy. Local complete response rate was 92% (483 cases), partial response rate was 8% (42 cases), non response rate was 0%, and the rate of progression was 0%. In 42 cases of local partial response in postoperative patients after 1 month assessed by radiology and criteria for radical RFA again, receiving completely RFA ablation again, completely response rate 100%. Total ablation times is 567 (for HCC patients with multinodular identified as a radical ablation session).

Follow-up was censored on 31st May 2016. Two patients were lost to follow-up as they did not respond to telephone follow-up. The data of these two patients were considered as censored data on their last available follow-up.

### Statistical analysis

Continuous variables were presented as mean ± standard deviation. Categorical data were presented as proportions. Survival was analyzed using the Kaplan-Meier method and the log-rank test. A multivariate Cox analysis was used to determine the independent factors associated with recurrence and survival. For multivariate analysis, continuous variables were categorized: age (≤50 vs. >50 years), tumor size (≤2 vs. 2–3 vs. 3–5 vs >5 cm), total bilirubin (<17 vs. 17–34 vs. >34 μmol/L), serum albumin (<33 vs. 33–37 vs. 37–41 vs. >41 g/L), platelet count (<50 vs. 50–90 vs. >90 × 10^9^/L), serum AFP (<50 vs. 50–100 vs. 100–400 vs. >400 ng/ml), HBV virus load (500–1000 vs. 1000–5000 vs. 5000–10,000 vs. 10000–100,000 vs. >100,000 copies/ml), and prothrombin time (<14.5 vs. >14.5 s). SPSS 16.0 (IBM, Armonk, NY, USA) was used for statistical analysis. Two-sided *P* values <0.05 were considered statistically significant.

## Results

### Characteristics of the patients

**Fig. 1:**
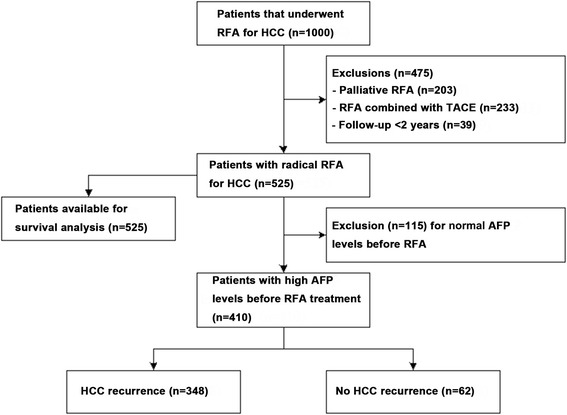
Patient flowchart

Figure 1 presents the patient flowchart. Among the 525 cases considered as having received curative ablation, there were 424 men and 101 women. The mean tumor size was 33.2 ± 12.2 (range, 7.4–72) mm. There were a total of 785 tumors for an average of 1.5/patient. All patients had either: (1) a single tumor of <5 cm; (2) ≤3 tumors of <3 cm; or (3) the sum of all tumors was <7.2 cm. Among the 525 patients, 66 had a recurrence after primary surgery. Liver function was Child-Pugh A in 513 patients and B in 12. All patients were ECOG 0.

Two patients died of leukemia, 13 patients were with upper gastrointestinal hemorrhage, 50 patients were with end-stage liver disease (including hepatic encephalopathy and infectious peritonitis after ascites), 8 patients received surgical treatments for recurrent tumor, and 12 patients received liver transplantation.

### Survival

**Fig. 2:**
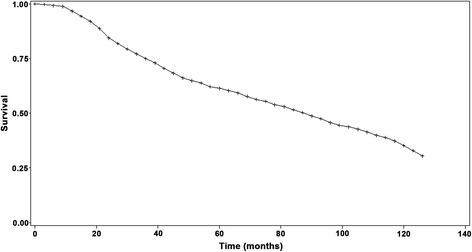
Overall survival of the 525 patients with HCC treated with radical RFA

Figure 2 presents the OS of the 525 patients. The 1-, 2-, 3-, 4-, 5-, 6-, 7-, 8-, 9-, and 10-year survival rates were 96, 84, 75, 61, 66, 60, 56, 51, 45, and 35%, respectively.

### Multivariate analysis

Table 1 presents the multivariate analysis for the risk of death after RFA for HCC. Results showed that tumor size (HR = 1.36, 95% CI 1.12–1.65, *P* = 0.002), albumin levels (HR = 0.76, 95% CI 0.65–0.91, *P* = 0.002), PT (HR = 2.18, 95% CI 1.54–3.10, *P* = 0.0001), and AFP levels (HR = 1.13, 95% CI 1.00–1.26, *P* = 0.049) were independently associated with mortality after RFA for HCC.

**Table 1:** Multivariate analysis for the risk of death after RFA for HCC

Variable	Hazard ratio (95% CI)	*P*
Tumor size	1.357 (1.119, 1.646)	0.0019
Albumin levels	0.764 (0.646, 0.905)	0.0018
Prothrombin time	2.183 (1.535, 3.104)	0.0001
AFP levels	1.134 (1.000, 1.258)	0.0492
Gender	0.932 (0.648, 1.340)	0.7039
Age	0.962 (0.637, 1.454)	0.8549
Number of tumors	1.027 (0.768, 1.373)	0.8595
HBV-DNA	0.963 (0.732, 1.266)	0.787

*95%CI* 95% CI confidence interval, *AFP* α-fetoprotein, *HBV* hepatitis B virus

### Risk of recurrence

The 410 patients with HCC and high AFP levels before RFA treatment were categorized into the recurrence and no recurrence groups. Table 2 presents the risk factors for recurrence. The multivariate analysis showed that tumor size (HR = 1.27, 95% CI 1.15–1.40, *P* = 0.0001) and HBV-DNA levels (HR = 7.70, 95% CI 3.57–16.63, *P* = 0.0001), AFP levels before treatment (HR = 2.172, 95% CI 1.256–3.756, *P* = 0.0055), and AFP response (HR = 4.722, 95% CI 1.053–21.184, *P* = 0.0427) were independently associated with the risk of recurrence of HCC after RFA.

**Table 2:** Univariate analysis for the risk of recurrence of HCC after RFA

Parameter	All patients (*n* = 420)	Patients with recurrence (*n* = 348)	Patients without recurrence (*n* = 62)	Univariate hazard ratio (95% confidence interval)	*P*	Multivariate hazard ratio (95% confidence interval)	*P*
Age	57.07 ± 9.31	58.32 ± 8.97	55.72 ± 9.52	0.992 (0.927, 1.063)	0.8288	0.992 (0.927, 1.06)	0.8288
Tumor size	32.44 ± 10.64	38.81 ± 9.47	25.49 ± 6.81	1.267 (1.148, 1.398)	0.0001	1.267 (1.148, 1.398)	0.0001
BPC	99.45 ± 59.81	95.05 ± 54.01	104.24 ± 65.46	1.000 (0.992, 1.008)	0.9839	1.000 (0.992, 1.008)	0.9839
BIL	21.05 ± 17.85	22.26 ± 16.58	19.74 ± 19.13	0.981 (0.917, 1.049)	0.5683	0.981 (0.917, 1.049)	0.5683
Albumin	36.91 ± 6.46	35.76 ± 6.14	38.15 ± 6.60	0.998 (0.880, 1.132)	0.974	0.998 (0.880, 1.132)	0.974
PT	14.53 ± 1.98	14.79 ± 2.11	14.24 ± 1.79	1.056 (0.694, 1.608)	0.7988	1.056 (0.694, 1.608)	0.7988
HBV-DNA	3.45 ± 1.77	4.62 ± 1.68	2.18 ± 0.57	7.699 (3.566, 16.625)	0.0001	7.699 (3.566, 16.625)	0.0001
AFP	1.367 ± 1.68	1.95 ± 1.69	0.73 ± 1.72	2.172 (1.256, 3.756)	0.0055	2.172 (1.256, 3.756)	0.0055
AFP response	0.531 ± 0.500	0.514 ± 0.502	0.549 ± 0.500	4.722 (1.053, 21.184)	0.0427	4.722 (1.053, 21.184)	0.0427

*BPC* blood platelet count, *BIL* total bilirubin, *PT* prothrombin time, *AFP* α-fetoprotein, *HBV* hepatitis B virus

### Mortality and recurrence

In order to reveal the median survival time differences between recurrence and non recurrence group in HCC with the high preoperative level AFP, exclude 115 cases with normal preoperative AFP level (21.9%, HCC patients with normal AFP), The 410 patients with HCC and high AFP levels before RFA treatment were categorized into the recurrence (348cases) and no recurrence groups (62 cases).

**Fig. 3:**
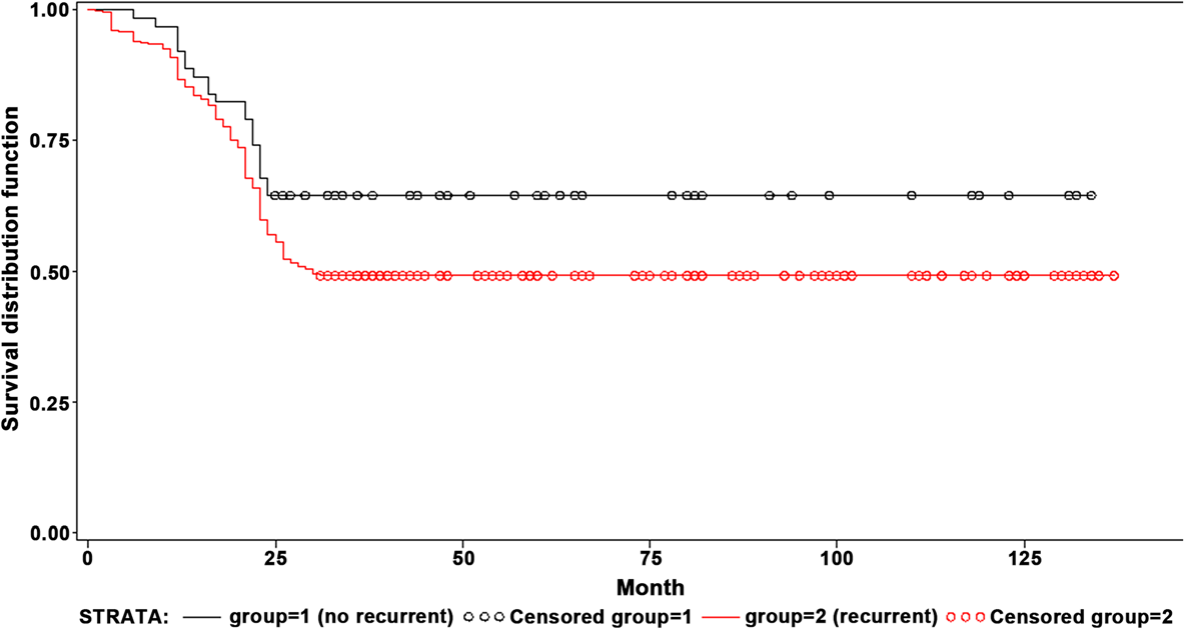
Survival of the patients with HCC treated with RFA according to the recurrence status

Figure 3 presents the survival of the patients according to the recurrence status. The median follow-up of the patients in the recurrence and no recurrence groups after RFA was 54 (95% CI 45–58) months and 62 (95% CI 48–80) months, respectively (log-rank, *P* = 0.04).

### Complications

In the present study, no death was attributed to RFA. Three patients had life-threatening complications requiring surgical interventions or blood transfusion. Among these three patients, one was with major thoracic hemorrhage, who recovered after the hematocele was cleared by surgery; one was with major abdominal hemorrhage, who recovered after percutaneous ultrasonography-guided gel injection to the bleeding site and blood transfusion; and the other patient was with subcutaneous hemorrhage accompanied with scrotum hematoma after RFA, who recovered after local pressuring and conservative treatment. In these three patients, the hemorrhage occurred at the early stage after RFA; their tumor size was relatively large, and the hemorrhage was caused by the repeated punctures during the overlapped ablation with a single electrode.

Three patients were with subcutaneous metastases. All three patients received repeated preoperative 18G automatic biopsies to obtain specimens for multiple times.

One patient suffered from hepatic infarction. This patient manifested postoperative fever and local pain. Ultrasound showed that there was no blood perfusion in the hepatic segments in the left liver lobe, and biopsy confirmed local infarction. The RFA probably damaged the small arteries, which induced ischemic necrosis of local intrahepatic bile ducts and gas accumulation.

Eight patients were with biloma at the site of RFA. In these patients, repeated punctures damaged the small bile duct and induced a bile pool at the site of necrosis area caused by RFA. After the formation of the biloma, the CEA and CA199 in these patients increased to different degrees. None of these patients required specific treatments. Imaging examinations 6–12 months after RFA showed that the bilomas were absorbed, and CEA and CA199 were both decreased.

No gastric-intestinal perforation was found in this study, which could be associated with the use of a straight rod ablation electrode, and the application of prophylactic measures for ascites during RFA. Two patients were with massive pleural effusion. One patient recovered after catheter drainage and the other recovered after fluid aspiration. Ascites were alleviated after conservative treatments.

All patients were with different degrees of postoperative local pain. The body temperature and liver enzyme level of all patients were elevated at different degrees 24 h after operation.

One patient was with minor local skin burn. This patient had received implantation of a metal graft due to femoral head necrosis. When the cool-tip skin electrode moved upward to the back, the overlapped ablation caused local skin reddening and swelling, as well as slight skin burn. The patient recovered without specific treatment.

## Discussion

The risk factors for recurrence and death after RFA for HCC remain poorly known. Therefore, this study aimed to study the 10-year OS of HCC treated by ultrasound-guided RFA and the risk factors for recurrence and death. Results showed that tumor size, albumin, PT, and AFP levels were independently associated with mortality after RFA for HCC, while tumor size, HBV-DNA, AFP levels before, and AFP response were independently associated with recurrence. Patients with recurrence had a poorer survival compared with those without.

The BCLC suggest that liver tumor diagnosis and treatment monitoring can be performed using liver ultrasound and especially CEUS [10, 19]. Leen et al. [20] and Minami et al. [21] advocated that CEUS is accurate and precise to determine liver tumor size and boundaries [20, 21]. BCLC also proposed that image-guided RFA can be used for minimally invasive thermal ablation for HCC and that the most convenient way to guide RFA is by ultrasound, which in itself is a non-invasive technique that has little effects on liver function and quality of life of the patients [10, 19].

In the present study, the 5- and 10-year survival rates were 66 and 35%, respectively, which were similar to that of previous studies (5-year 40.0–60.2%, 10-year 27.3%) [5, 22–24]. Of course, OS is related to the immune status, tumor size, HBV DNA load, liver function, systemic diseases, and margins, as well to complete or partial ablation by RFA, and differences in these parameters may be responsible for differences among studies [5, 22–24]. Other factors are also associated with the prognosis of HCC after RFA, such as margin status, immune response, immune escape, and VEGF secretion [25].

In order to improve the OS after local tumor ablation, it is a necessity to identify the risk factors for OS and recurrence. The present study showed that tumor size, albumin, prothrombin time, and α-fetoprotein levels were independently associated with mortality after RFA for HCC, while tumor size and HBV-DNA were independently associated with recurrence. These results are similar to those of previous studies. Indeed, Shiina et al. [5] showed that age, anti-HCV, Child-Pugh class, tumor size, serum DCP levels, and AFP-L3 levels were associated with survival. N’Kontchou et al. [23] showed that PT and AFP were associated with survival, while multinodular HCC and AFP levels were associated with recurrence after RFA. PT is a vitamin K-dependent coagulation factor synthesized by the liver, and is a reflection of the liver synthetic function. Liver reserve function will directly influence survival. Hepatitis induced by HBV infection is an important cause of liver function damage and long-term infection leads to liver cell damage, fibrosis, and hepatic reserve function decline [10, 19].

Liver tumor recurrence involves complex mechanisms and studies show that after HCC resection, the overall 5-year postoperative recurrence rate is 50–81% [26–28]. The present study showed that 2 years after treatment, the recurrence rate was 21%, among which 93% were regional recurrence away from the previous treatment site and 7% were local progression. In the present study, the recurrence was associated with poorer survival compared with patients without recurrence, suggesting that patients with recurrence have more active disease that may metastasize or continue deteriorating liver function. Kang et al. [29] showed that periportal tumor location and age were associated with recurrence, and that recurrence affected OS.

The present study is not without limitations. The sample size was relatively limited and from only four centers. The selection criteria may have introduced a selection bias, limiting the generalizability of the results. Only a limited number of factors were studies. Future studies could explore additional factors that could be involved in survival and recurrence, such as inflammatory markers. Additional studies are still necessary to adequately predict the prognosis of HCC after ultrasound-guided RFA.

## Conclusions

In conclusion, tumor size, albumin, PT, AFP levels were independently associated with mortality after ultrasound-guided RFA for HCC, while tumor size, HBV-DNA, AFP levels before treatment, and AFP response were independently associated with recurrence. Patients with recurrence had a poorer survival compared with those without. Preoperative high level of AFP HCC patients with postoperative recurrence is an important clinical poor indicator in the median survival time.

## Abbreviations

AFP: α-fetoprotein
BIL: Total bilirubin
BPC: Blood platelet count
CBC: Complete blood count
CEUS: Contrast-enhanced ultrasound
CT: Computed tomography
DCP: Des-gamma-carboxy-prothrombin
HBV: Hepatitis B virus
HCC: Hepatocellular carcinoma
MRI: Magnetic resonance imaging
OS: Overall survival
PET: Positron emission tomography
PT: Prothrombin time
RFA: Radiofrequency ablation
